# Particle Focusing under Newtonian and Viscoelastic Flow in a Straight Rhombic Microchannel

**DOI:** 10.3390/mi11110998

**Published:** 2020-11-11

**Authors:** Joo-Yong Kwon, Taehoon Kim, Jungwoo Kim, Younghak Cho

**Affiliations:** 1 Department of Mechanical Design and Robot Engineering, Seoul National University of Science & Technology, 232 Gongneung-ro, Nowon-gu, Seoul 01811, Korea; wock2003@naver.com (J.-Y.K.); tkim99@seoultech.ac.kr (T.K.); kimjw@seoultech.ac.kr (J.K.); 2 Department of Mechanical System Design Engineering, Seoul National University of Science & Technology, 232 Gongneung-ro, Nowon-gu, Seoul 01811, Korea

**Keywords:** rhombic microchannel, elasto-inertial particle focusing, Newtonian fluid, viscoelastic fluid

## Abstract

Particle behavior in viscoelastic fluids has attracted considerable attention in recent years. In viscoelastic fluids, as opposed to Newtonian fluids, particle focusing can be simply realized in a microchannel without any external forces or complex structures. In this study, a polydimethylsiloxane (PDMS) microchannel with a rhombic cross-sectional shape was fabricated to experimentally investigate the behavior of inertial and elasto-inertial particles. Particle migration and behavior in Newtonian and non-Newtonian fluids were compared with respect to the flow rate and particle size to investigate their effect on the particle focusing position and focusing width. The PDMS rhombic microchannel was fabricated using basic microelectromechanical systems (MEMS) processes. The experimental results showed that single-line particle focusing was formed along the centerline of the microchannel in the non-Newtonian fluid, unlike the double-line particle focusing in the Newtonian fluid over a wide range of flow rates. Numerical simulation using the same flow conditions as in the experiments revealed that the particles suspended in the channel tend to drift toward the center of the channel owing to the negative net force throughout the cross-sectional area. This supports the experimental observation that the viscoelastic fluid in the rhombic microchannel significantly influences particle migration toward the channel center without any external force owing to coupling between the inertia and elasticity.

## 1. Introduction

Particle manipulation in microfluidic channels has been widely applied in the biological, medical, chemical, and environmental fields owing to their capacity for high efficiency, high accuracy, and fast processing with the additional advantages of low cost and reduced sample consumption [1,2,3,4,5]. A variety of microfluidic devices for particle focusing, which are based on active and passive methods, have been widely researched and developed. Inertial microfluidics as a representative passive method has been extensively studied and employed to accomplish particle ordering, focusing, and separation [6,7,8] in a Newtonian fluid such as deionized (DI) water and phosphate-buffered saline (PBS) solution. Although this method has certain advantages such as that an external force is not required, the device is easy to operate, and the microchannel structure is simple, the disadvantage is that particles may be focused in multiple linear streams (rather than a single stream) because of the inertial forces in a Newtonian fluid.

Recently, particle manipulation in a viscoelastic non-Newtonian fluid using microfluidic devices has generated widespread interest because of the ability of these fluids to achieve superior 3D particle focusing in simple straight channels without the need for labeling or the application of external force fields [9,10]. Particle migration in a viscoelastic fluid differs completely from that in a Newtonian fluid. Particles in Newtonian fluids experience two different types of forces: Shear gradient force and wall-repulsion force. On the other hand, particle behavior in non-Newtonian fluids is affected by fluid elasticity in addition to inertial forces, thus particle migration in viscoelastic fluids has given rise to highly interesting and useful phenomena [11].

Because of their simple structure, straight microchannels are widely used to study particle focusing in viscoelastic fluids [10,11,12,13]. In addition, the sample throughput can be further improved as a result of the excellent parallelization of the extremely simple design when using a straight microchannel. Particle focusing in a straight microchannel is known to be greatly influenced by the shape of the cross-section of the microchannel [10]. Yang et al. demonstrated a method for sheathless particle focusing, known as elasto-inertial particle focusing, in a 50-μm wide straight square microchannel by using the synergetic effect of inertia and viscoelasticity in a viscoelastic fluid consisting of an aqueous solution of poly(ethylene oxide) (PEO, 0.05 wt%) [11]. The migration and single-line focusing of microspheres in the microchannel flow of viscoelastic fluids were investigated by Seo et al. who used a holographic microscopy technique [10]. They succeeded in obtaining information on the 3D positions of particles flowing in a square microchannel according to the blockage ratio and flow rate. Liu et al. presented sheathless, label-free, and size-based particle/cell separation in a simple straight rectangular microchannel utilizing viscoelastic focusing [12]. The large components were focused near the sidewalls whereas the small components were focused along the centerline. Kang et al. reported that the strong elastic effects of a very dilute DNA solution (0.0005 (w/v)%) flowing through a circular microchannel led to lateral particle motion toward the centerline [13]. In their work, they accomplished highly tunable three-dimensional (3D) particle focusing over a wide range of flow rates in a simple straight microchannel using the non-Newtonian elastic properties of a DNA-laden fluid.

Thus far, particle behavior in inertial and elasto-inertial microfluidics and the application thereof to the above-mentioned fields has been mainly achieved with microchannels with a rectangular cross-sectional shape because of the limitation imposed by the classical soft lithography technique. More recently, owing to recent improvements in the microfabrication technique, many researchers were able to fabricate microchannels with non-rectangular cross-sectional shapes such as circular, semi-circular, triangular, and trapezoidal by combining various mold fabrication techniques with the conventional polydimethylsiloxane (PDMS) cast molding method. Kim et al. fabricated semi-circular channel molds by reflowing the rectangular photoresist and triangular channel molds with the use of an anisotropically etched Si wafer and demonstrated inertial focusing in a Newtonian fluid [14]. Microchannels with triangular and trapezoidal cross-sections were fabricated to study the inertial focusing of microparticles and manipulate them in non-rectangular cross-sectional channels [15,16]. Tang et al. proposed a novel 3D-printed mold-removal method to fabricate microchannels with semi-elliptical and triangular cross-sections in which the mechanisms of elasto-inertial focusing were explored [17]. In our previous paper, we proposed a novel yet simple method to fabricate microchannels with various cross-sectional shapes, such as rhombic, pentagonal, hexagonal, and with the shape of a parallelogram. The method is based on the basic microelectromechanical systems (MEMS) processes, viz. photolithography, reactive ion etching (RIE) and anisotropic potassium hydroxide (KOH) wet etching followed by self-alignment between the Si structure and PDMS mold [18].

Apart from the experimental studies, several numerical studies were carried out to evaluate particle behavior in microchannels with various cross-sections under Newtonian or non-Newtonian fluid flow. Modeling particle fluid interactions using a direct numerical simulation (DNS) method, Raoufi et al. studied the effect of the channel geometry and corner angle on particle focusing in viscoelastic solutions [19]. Additionally, numerical simulations were conducted on viscoelastic fluid flows in straight ducts with different cross-sections, allowing the origin of secondary flows and the influence of material parameters and the geometrical configuration of the flow passage to be numerically investigated [20]. They studied the secondary flows of viscoelastic fluids in straight ducts by embedding the Giesekus constitutive model into FLUENT by using a user-defined function (UDF). Yu et al. investigated elasto-inertial particle migration in the rectangular channel flow of an Oldroyd-B viscoelastic fluid by means of three-dimensional DNS [21].

In this paper, we present a simple method to fabricate a PDMS rhombic microchannel. We carried out experiments to examine inertial and elasto-inertial particle focusing in the rhombic microchannel. The particle migration in Newtonian and non-Newtonian fluids in the rhombic microchannel were compared in terms of the flow rate and particle size, and their effect on the particle focusing position and focusing width were also investigated. The results showed that single-line particle focusing occurred along the centerline of the microchannel in the non-Newtonian fluid, unlike the double-line particle focusing in the Newtonian fluid over a wide range of flow rates. Our design may serve as a promising microfluidic pretreatment platform for flow cytometry.

## 2. Materials and Methods

### 2.1. Fabrication of Microchannel with Rhombic Cross-Section

**Figure 1:**
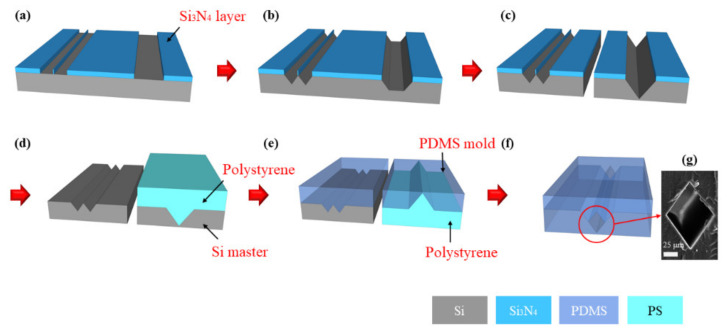
Schematic three-dimensional (3D) view of fabrication processes of the microchannel with a rhombic cross-sectional shape (**a**–**f**) and cross-sectional scanning electron microscope (SEM) image of fabricated rhombic microchannel (**g**). (**a**) Si_3_N_4_ layer patterning; (**b**) Si wet etching; (**c**) dicing and additional wet etching of Si channel; (**d**) hot-embossing polystyrene(PS) after removal of Si_3_N_4_ layer; (**e**) polydimethylsiloxane (PDMS) molding; (**f**) plasma bonding of PDMS; (**g**) PDMS rhombic microchannel with a side length of 73.5 μm.

The fabrication process of microchannels with a rhombic cross-sectional shape is illustrated in Figure 1. The microchannels were formed using the basic MEMS processes, i.e., photolithography, RIE, and anisotropic KOH wet etching following the conventional soft lithography technique. The typical fabrication processes are as follows: (i) A thin film of Si_3_N_4_ with a layer thickness of 1000 Å was deposited on a (100) single-crystal Si wafer using low-pressure chemical vapor deposition (LPCVD) and patterned by photolithography and RIE; (ii) the Si wafer was anisotropically etched with KOH solution at 70 °C (additional wet etching of the Si microchannels was performed after dicing the Si wafer); (iii) a PDMS (Dow Corning, Midland, MI, USA) microchannel was replicated from a polystyrene (PS) mold created from the Si microchannel; (iv) the PDMS mold was replicated from the Si master; (v) the PDMS microchannel and the PDMS mold were self-aligned and bonded by O_2_ plasma. A small amount of methanol (or DI water) was sprayed between the PDMS microchannel and the PDMS mold to facilitate self-alignment. Finally, the methanol was evaporated on a hot plate to complete the formation of the microchannels which were composed of PDMS only.

### 2.2. Sample Preparation and Experimental Setup

DI water was used as the Newtonian solution, and an aqueous solution of PEO was selected as the viscoelastic solution. The PEO solution (M_w_ = ~2 MDa, Sigma-Aldrich, Saint Louis, MO, USA) was prepared by dissolving PEO in DI water to obtain a 0.05 wt% PEO (PEO500) solution. Then, fluorescent polystyrene (PS) particles (Thermo Scientific inc., Fremont, CA, USA) with particle sizes of 5 and 13 μm were added to these experimental solutions (0.05–0.1 wt% concentration), respectively. To prevent particle aggregation in the particle migration experiments, the surfactant Tween 20 (Sigma-Aldrich, Saint Louis, MO, USA) was added to the suspensions at 0.1 wt%. The estimated mean viscosity of the PEO500 solution was approximately 1.85 mPa∙s (DI water: 0.89 mPa∙s) [22,23,24]. Its relaxation time was estimated from the previous empirical relaxation time (*λ*_e_), which was also measured with a capillary breakup extensional rheometer (CaBER) [22,23,24]. The relaxation time for the PEO500 solution was estimated to be 3.78 ms on the basis of the empirical formula [24].

The particles in the microchannel were observed using inverted optical microscopes (BX-60, Olympus, Tokyo, Japan). The flow rates were controlled with a syringe pump (LEGATO 111, KD Scientific Inc., Holliston, MA, USA) and the images were captured 4 cm downstream from the inlet using CMOS camera (Touptek Photonics Co., Ltd, Hangzhou, China). To allow the particle focusing behavior to be precisely analyzed, bright-field images and fluorescent images were captured with exposure times of 20 ms and 500 ms, respectively. All the analyses and post-processing of the captured images were performed using the open-source ImageJ software. In this work, the fluorescent intensity profile across the channel was extracted and fitted with a Gaussian distribution.

### 2.3. Simulation for Viscoelastic Flow

In addition to the experimental work, numerical simulation was also conducted using a finite-volume-based flow solver (ANSYS Fluent). The objective of the numerical approach was to shed light on the influence of the viscoelastic fluid on particle migration particularly in a rhombic microchannel. To this end, in the present numerical study, the equilibrium position of inertial particles, induced by various forces acting on the particles in a microchannel with a rhombic cross-sectional shape, was investigated. Similar to the experiment, two particle diameters (i.e., d = 5 and 13 μm) and two representative flow rates (i.e., 1 and 10 μL/min) were considered in this numerical simulation to highlight the viscoelastic effect on inertial focusing.

**Figure 2:**
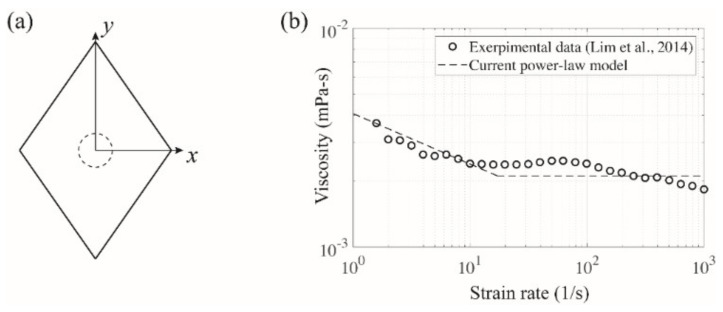
(**a**) Cross-sectional shape of the rhombic microchannel utilized in the numerical simulation. The dashed circle represents a spherical particle located in the center of the channel. (**b**) Power-law model based on the experimental data [23] to simulate the viscosity of the PEO500 solution.

Figure 2a presents the rhombic cross-sectional area considered herein, which has the same physical dimensions as those in the experiment. The axial length of the channel was set to 1 mm for convenience of calculation. A uniform velocity and uniform pressure were used for each inlet and outlet. The corresponding channel Reynolds numbers (Re=UinDh/ν, where $U_{in}$, $D_{h}$, and ν denote the inlet velocity, the hydraulic diameter of the channel, and the kinematic viscosity of the working fluid, respectively) are 0.17 and 1.68 for flow rates of 1 and 10 μL/min, respectively. The particle Reynolds number (Rep=Red/Dh2, where *d* represents the particle diameter) is thus in the range of $O\left(10^{-4}\right)-O\left(10^{-2}\right)$. The flow and particle parameters in this numerical study are summarized in Table 1. In this simulation, an unstructured non-uniform grid was employed to discretize the entire computational domain, and the total number of grid points was approximately 500,000 in all cases. In particular, the grid resolution around the spherical particle was set to Δ/d=0.1 for both of the particle sizes (5 and 13 μm) for consistency.

Owing to the nature of the viscoelastic fluid (PEO500) considered herein, it is of particular importance to model the viscosity with respect to a range of shear rates in this numerical simulation. Based on the experimental data that were obtained by employing the same working fluid [23], a power-law model was utilized to simulate the viscosity of the non-Newtonian fluid used in this work as shown in Figure 2b. It should be noted that this power-law model has a lower limit for $\dot{\gamma }>18$, whereas the experimental data exhibit a second decreasing tendency in viscosity followed by a certain plateau for $10<\dot{\gamma }<100$. Thus, the lower limit in this model was considered to be the average between the viscosity corresponding to the plateau and that at $\dot{\gamma }=1000$ in the experimental data with the aim of minimizing the numerical uncertainty.

A single rigid spherical particle was specified at a fixed location in the cross-sectional area of the rhombic microchannel (see Figure 2a). Multiple positions along the line of symmetry (i.e., the *x-* and *y-*axes herein) were chosen to examine the variation in the net lift force acting on the particle with respect to the channel center. It is noteworthy that the net lift force in this study was directly computed by ANSYS Fluent instead of calculating and combining individual force terms such as the wall-induced lift force, shear-gradient lift force, and viscoelastic force. The Magnus effect, attributed to the particle rotation and its corresponding pressure difference, was not considered in the net lift force because the spherical particle herein was fixed as an irrotational rigid body in the microchannel. Despite its significant contribution to the inertial particle focusing, the Magnus effect can be ignored when the particle Reynolds number is much smaller than 0.1 [25] as is the case for the current simulation regime. Thus, the analysis of the net lift force in this study allows us to estimate the mechanism of the inertial particle focusing and its resulting equilibrium position based on the magnitude and slope of the net lift force. Furthermore, the effect of the viscoelastic fluid on particle migration was expected to be highlighted upon comparison with an experimental and numerical study with the same channel geometry but a different working fluid (Newtonian DI water) [26]. This is discussed in great detail in Section 3 “Results and Discussion.”

## 3. Results and Discussion

The side length of the fabricated rhombic microchannel was 73.5 μm and its hydraulic diameter (D_h_) was calculated to 69.4 μm.

First, we investigated the particle focusing in the Newtonian fluid and then observed the migration of particles in the non-Newtonian viscoelastic fluid in the rhombic microchannel. Reynolds number (Re) is a dimensionless number indicating the relative importance of the inertial effect and viscous effect Equation (1). In a non-Newtonian viscoelastic fluid, particles are affected by an additional elastic force, which is determined by the intrinsic properties of the medium solution. The elastic effects of a non-Newtonian fluid in the channel can be characterized by the Weissenberg number (Wi) which is a dimensionless number Equation (2).

In the case of particle focusing in the non-Newtonian viscoelastic fluid, the fluid elasticity is a significant factor that governs the focusing behavior. That is, the number of particle focusing points can be determined by the Reynolds number (Re) and Weissenberg number (Wi) [2]. Therefore, the fluid elasticity (El) is defined as the ratio of Weissenberg number to the Reynolds number Equation (3). The comparison of these two dimensionless numbers makes it possible to identify the major force responsible for the particle focusing phenomena. For a rhombic microchannel, these parameters can be defined as follows [11]:

Re = *ρ*V_m_ D_h_ /μ = *ρ*;Q sin 2θ/μD_h_ (1)

W_i_ = λγ̇ = 2λV_m_/w = 2λQ sin 2θ ⁄ D_h_^3^ (2)

El = Wi/Re = 2λμ/𝜌D_h_^2^ (3)

where 𝜌 is the fluid density, *V_m_* is the characteristic velocity, D_h_ is the hydraulic diameter, *μ* is the mean viscosity, λ is the relaxation time of the polymer solution, 𝛾̇ is the shear rate, *w* is the microchannel width (same as the hydraulic diameter in case of a rhombus), Q is the flow rate, and *θ* is 54.7º (the etch angle between the (111) and (100) planes of Si).

### 3.1. Particle Focusing in Rhombic Microchannel

**Figure 3:**
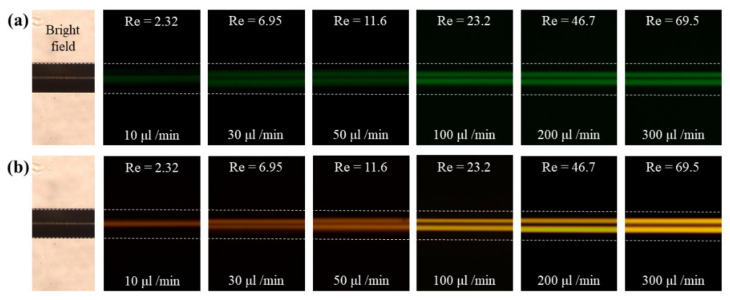
Fluorescence image of the flow through the rhombic microchannel in the Newtonian fluid at various flow rates: (**a**) 5 μm particles and (**b**) 13 μm particles.

Figure 3 presents the focusing of particles in the microchannel with the rhombic cross-sectional shape at various flow rates and for different particle sizes in DI water. The dotted lines illustrate the ends of the rhombic microchannel, which were obtained from the bright-field images under the same experimental conditions. As shown in Figure 3a, the particles with a 13 μm diameter are distributed and scattered around the center of the rhombic microchannel for low flow rates of less than 10 μL/min because the inertial force is insufficiently strong to drive all the particles toward their equilibrium positions. As the flow rate increases to above 100 μL/min, two inertial focusing points were clearly observed as a result of the strengthening inertial forces within the Newtonian fluid [18]. That is, the equilibrium positions are created by the balance of the two lift forces (the shear gradient lift force and wall lift force), which are the two dominant forces that govern particle migration in an inertial dominant flow without elasticity (Re > 0 and Wi ≈ 0) [6]. The rhombic microchannel has two inertial focusing points near the corner of the microchannel that forms an obtuse angle, whereas a square microchannel has four inertial focusing points at the center of each side of the channel wall [18,26]. Particles could be efficiently focused and separated at a relatively low Reynolds number in a rhombic microchannel compared with a square microchannel [26]. On the other hand, under the same flow conditions, small particles with a diameter of 5 μm exhibit wider focusing bands than the large particles with a diameter of 13 μm (Figure 3) because of the difference in the inertial force exerted on these particles with different sizes, which is proportional to a fourth of the size of the particle [10].

**Figure 4:**
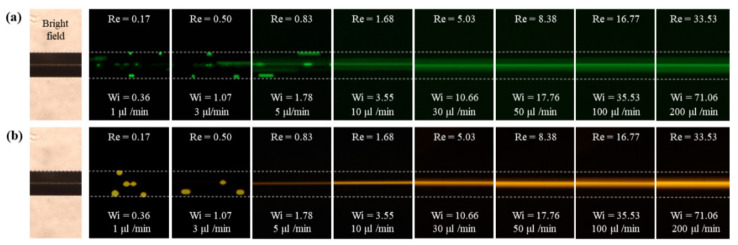
Fluorescence image of the rhombic microchannel containing the viscoelastic fluid (consisting of an aqueous solution of PEO500) at various flow rates: (**a**) 5 μm particles; (**b**) 13 μm particles.

The particle behavior in the non-Newtonian viscoelastic fluid differs entirely from that in the Newtonian fluid. Figure 4 shows the particle focusing for the various flow rates and the two different particle sizes in the PEO500 solution. The blockage ratio (β = d/D_h_) of the particle diameter (d) to the hydraulic diameter of the channel (D_h_) is an important parameter that determines the elasto-inertial particle focusing behavior [10]. To investigate the effect of the flow rate and blockage ratio on the elasto-inertial focusing, particles suspended in PEO500 solution were injected at flow rates ranging from 1 to 200 μL/min. At low flow rates (Re ≈ 0 and Wi > 0) which correspond to a negligible inertial force and relevant elastic force, the equilibrium points of particles with a diameter of 5 μm (β = 0.07) were located at the channel center and corners up to the flow rate of 5 μL/min; thus, the particles moved either along the center or in the corners of the rhombic microchannel. As the flow rate increased to 10 μL/min (Re > 0 and Wi > 0), the particles were focused at the center of the microchannel owing to the non-negligible inertial force and relevant elastic force. In other words, elasto-inertial particle focusing occurred, which is the result of competition between the inertial lift force and elastic force in a viscoelastic non-Newtonian fluid flowing through a straight microchannel [10,11]. As shown in Figure 3b, particle migration and focusing were more distinct at a high blockage ratio (β = 0.19) because the force on the particle induced by normal stress is proportional to the cube of the particle diameter [10].

**Figure 5:**
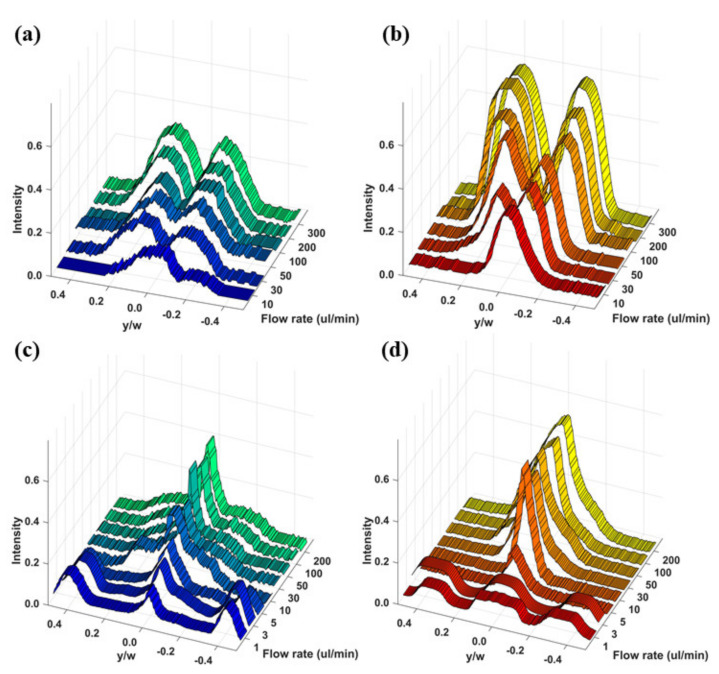
Fluorescence intensities (top view) according to various flow rates: (**a**) 5 μm and (**b**) 13 μm particles in the Newtonian fluid, (**c**) 5 μm and (**d**) 13 μm particles in the viscoelastic fluid.

The normalized fluorescent intensity profiles illustrating the particle distribution at different flow rates and for the two different particle diameters are shown in Figure 5. Tighter focusing was observed at a high blockage ratio (β = 0.19) for both Newtonian and non-Newtonian fluids. For the Newtonian fluid flow, as the flow rate increased, most particles migrated toward two points near the obtuse angle corner of the rhombic microchannel. In the case of the flow rate of 100 μL/min, approximately 65% of the particles were focused at two equilibrium points for a low blockage ratio (β = 0.07), whereas more than 87% of the particles were focused at these points for a high blockage ratio (β = 0.19). This means that the lateral migration of the particles in a Newtonian fluid is mainly governed by the inertial force, which is affected by the particle size [6,10].

For the non-Newtonian fluid flow, particles were observed to focus at three different positions in the rhombic microchannel, depending on the flow rate, which was also observed for the square microchannel [10]. That is, when the flow rate increased from 1 to 10 μL/min, the number of focusing positions decreased from five to one as a result of the purely elastic regime transforming into an elasto-inertial regime. On the other hand, the focused particles were slightly dispersed around the centerline when the flow rate increased to 100 μL/min because of the relatively strong inertial force prevailing at the high flow rate [11]. At the flow rate of 10 μL/min, approximately 70% of the particles were focused at the center of the microchannel for a low blockage ratio (β = 0.07), whereas more than 90% of the particles were focused here for a high blockage ratio (β = 0.19). This means that the lateral migration of particles in a non-Newtonian fluid are governed by both the inertial force and elastic force and that large particles are subjected to a stronger lateral force resulting from the inertial effect than small particles [6,10].

### 3.2. Simulation Results

**Figure 6:**
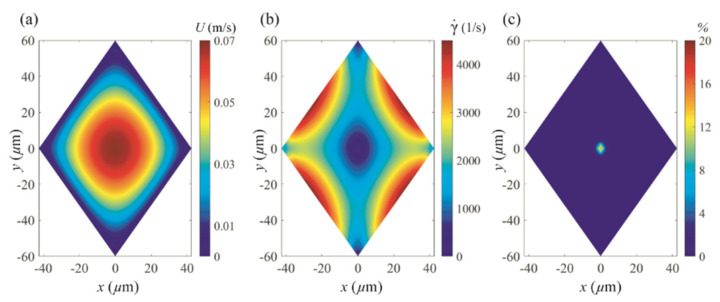
Simulation results of the (**a**) streamwise velocity magnitude (m/s), (**b**) shear rate (1/s), and (**c**) percent discrepancy in the shear rate between the current viscoelastic fluid (PEO500 solution) and DI water in a cross section of the rhombic microchannel at 10 μL/min.

Figure 6a,b show the magnitude of the velocity and shear rate obtained from the numerical simulation with the viscoelastic fluid at the flow rate of 10 μL/min, respectively. In the average sense, the magnitude of the velocity reaches a local maximum in the center of the channel and gradually decreases toward the wall as expected. The shear rate is a differential of the flow velocity described as $\dot{\gamma }={\left[{\left(\partial u/\partial x\right)}^{2}+{\left(\partial u/\partial y\right)}^{2}\right]}^{1/2}$, and its contour map, shown in Figure 6b, presents a single region in the center of the channel in which the shear rate is zero and four regions in which the shear rate approaches zero in each corner. These regions are closely related with inertial focusing as particles are likely to migrate toward zero shear rate regions when the second normal stress difference in a viscoelastic fluid is negligible [27,28]. Not only that, the shear rate is also associated with the inertial lift force caused by the shear gradient and wall interaction [29]. Therefore, the percent discrepancy in the shear rate between the present non-Newtonian working fluid and DI water at the same flow rate (i.e., 10 μL/min) is defined as follows:

(4) $$\Delta \dot{\gamma }\left(\%\right)=\frac{\left|{\dot{\gamma }}_{visc}-{\dot{\gamma }}_{water}\right|}{{\dot{\gamma }}_{visc}}\times 100$$

where ${\dot{\gamma }}_{visc}$ and ${\dot{\gamma }}_{water}$ denote the shear rates for the present viscoelastic fluid and DI water, respectively. This discrepancy constitutes the difference in the shear rate between the situations in which these two fluids are used in this work. As shown in Figure 6c, it is clear that, at the same flow rate, the shear rate of the current viscoelastic fluid and that of DI water is almost the same except for the center of the channel. This means that the viscoelastic effect on inertial focusing can be isolated upon comparison with the particle migration pattern of DI water for a microchannel with the same geometry and at the same flow rate.

**Figure 7:**
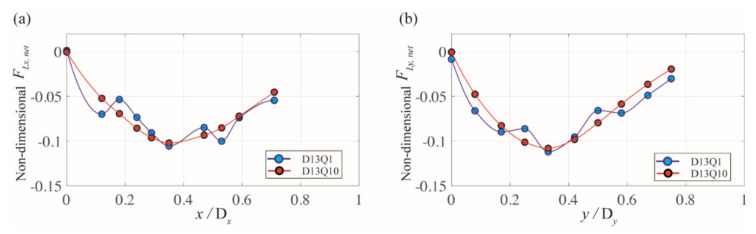
Variation in the (**a**) *x*-directional and (**b**) *y*-directional net force exerted on a 13-μm particle along the line of symmetry of the rhombic microchannel. The blue and red symbols represent the flow rates of 1 and 10 μL/min, respectively.

Figure 7a,b present the variation in the *x*- and *y*-directional net force ($F_{L,\;net}$) for 13-μm particles along the line of symmetry. This net force is normalized with $\rho_{f}U_{cl}^{2}d^{4}/D_{h}$, where $\rho_{f}$ and $U_{cl}$ denote the fluid density and the centerline velocity of the channel. The blue (Case D13Q1) and red (Case D13Q10) symbols represent flow rates of 1 and 10 μL/min, respectively. The value of $F_{L,\;net}$ was not computed for *x/*D*_x_* and *y/*D*_y_* values higher than 0.7 simply because the spherical particle starts to touch the channel wall.

In Figure 7, the positive and negative values of $F_{L,\;net}$ indicate the direction of the force toward the channel center and the corner bisector. For the flow rate of 10 μL/min (Case D13Q10), the net forces in both directions are mainly negative with one zero point at the channel center, which constitutes the opposite behavior compared with that in Newtonian fluids [12,26,30]. This indicates that particles suspended in the rhombic channel are likely to migrate toward the channel center. Therefore, the channel center is a particle focusing position as a result of the inertial lift force balancing the viscoelastic force and vice versa. The experimental results shown in Figure 4b support this notion as a single train of fluorescent particles resides at the channel center at the same flow rate. When the Reynolds number decreases (Case D13Q1) in Figure 7, the variation in the net forces starts fluctuating for both lines of symmetry, while their values remain negative. In this case, the particles are also supposed to drift toward the channel center where $F_{L,\;net}\sim \;0$. However, the experimental result under the same flow conditions (see Figure 4b) shows that particles seem to remain at rest owing to the lack of fluid inertia. This difference in the behavior from that observed experimentally is attributed to the fact that the numerical simulation computes the net lift force based on a fixed spherical particle. Therefore, the net lift forces herein only indicate the direction of the force but not the actual particle migration, which would have to take the fluid inertia into account.

**Figure 8:**
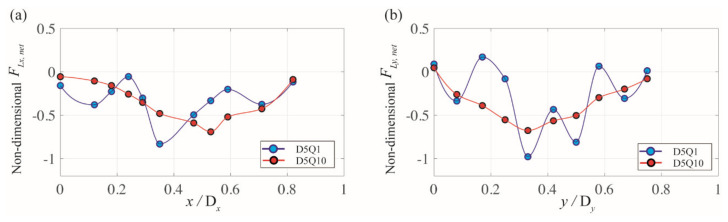
Variation in the (**a**) *x*-directional and (**b**) *y*-directional net force exerted on the 5-μm particle along the line of symmetry of the rhombic microchannel. The blue and red symbols represent flow rates of 1 and 10 μL/min, respectively.

The same information but for the 5-μm particle is plotted in Figure 8. Particularly, for the flow rate of 10 μL/min (Case D5Q10), the pattern of the *x*-directional net force ($F_{Lx,\;net}$) is different from that for Case D13Q10. In Figure 8a, the value of $F_{Lx,\;net}$ (red symbols) remains near zero and gradually increases as the distance from the channel center increases. In addition, the lateral position of the local minimum is shifted toward the corner (i.e., *x/*D*_x_*~0.55) as compared with that of Case D13Q10 (i.e., *x/*D*_x_*~0.34). Therefore, in this case, the particles continue to migrate toward the channel center as a single equilibrium position forms at the center. However, the particles may be less strongly clustered at the inertial focusing position owing to the relatively smaller magnitude of $F_{Lx,\;net}$ and a shifted local minimum in comparison with that of Case D13Q10. The experimental results in Figure 4a confirm this notion as the fluorescence image of the 5-μm particles at the flow rate of 10 μL/min shows a wider focusing band than that of the 13-μm particles at the same flow rate.

## 4. Conclusions

In this work, we fabricated a PDMS microchannel with a rhombic cross-sectional shape using simple MEMS processes, hot-embossing, micromolding, and self-alignment of the PDMS molds. We carried out experiments to investigate the inertial and elasto-inertial particle focusing behavior in a straight rhombic microchannel using Newtonian and non-Newtonian fluids. Various flow rates and two different particle sizes were used, and their effect on the particle focusing position and focusing width were investigated. The experimental results showed that single-line particle focusing occurred along the centerline of the microchannel for the non-Newtonian fluid, contrary to the double-line particle focusing in the Newtonian fluid at the various flow rates. That is, the combined effects of inertia and elasticity realized elasto-inertial particle focusing at the center of the rhombic microchannel in the absence of any external force. Furthermore, we numerically simulated the elasto-inertial focusing to investigate the combined effect of the cross-sectional geometry and viscoelasticity on the focusing phenomenon. The experimental results for the viscoelasticity-induced migration of particles agreed quantitatively with the simulation results.

This PDMS microfluidic device with the rhombic microchannel is expected to serve as a promising candidate for a microfluidic pretreatment platform for flow cytometry as well as a cost-effective and disposable device for particle/cell separation and sorting.

## Figures and Tables

**Table 1:** Flow and particle parameters in the numerical simulation.

Case	*d* (μm)	Flow Rate (μL/min)	*U*_in_ (m/s)	Re	Re*_p_*
D5Q1	5	1	$3.3\times 10^{-3}$	0.17	$8.7\times 10^{-4}$
D5Q10	5	10	$3.3\times 10^{-2}$	1.68	$8.7\times 10^{-3}$
D13Q1	13	1	$3.3\times 10^{-3}$	0.17	$5.9\times 10^{-3}$
D13Q10	13	10	$3.3\times 10^{-2}$	1.68	$5.9\times 10^{-2}$
